# Immunogenicity and safety of a tetravalent dengue vaccine in dengue-naïve adolescents in Mexico City

**DOI:** 10.26633/RPSP.2021.67

**Published:** 2021-06-11

**Authors:** Shibadas Biswal, Jorge Fernando Mendez Galvan, Mercedes Macias Parra, Juan-Francisco Galan-Herrera, Monica Belisa Carrascal Rodriguez, Esteban Patricio Rodriguez Bueno, Manja Brose, Martina Rauscher, Inge LeFevre, Derek Wallace, Astrid Borkowski

**Affiliations:** 1 Takeda Vaccines Inc. Boston United States of America Takeda Vaccines Inc., Boston, United States of America.; 2 Hospital Infantil de México Federico Gómez Mexico City Mexico Hospital Infantil de México Federico Gómez, Mexico City, Mexico; 3 Instituto Nacional de Pediatría Mexico City Mexico Instituto Nacional de Pediatría, Mexico City, Mexico; 4 Instituto Politécnico Nacional Mexico City Mexico Instituto Politécnico Nacional, Mexico City, Mexico; 5 CAIMED Investigación en Salud Mexico City Mexico CAIMED Investigación en Salud, Mexico City, Mexico; 6 Mexico Center for Clinical Research Mexico City Mexico Mexico Center for Clinical Research, Mexico City, Mexico; 7 Takeda Pharmaceuticals International AG. Zurich Switzerland Takeda Pharmaceuticals International AG., Zurich, Switzerland

**Keywords:** Vaccines, adolescents, immunogenicity, safety, dengue, Mexico, Vacunas, adolescentes, inmunogenicidad, seguridad, dengue, México, Vacinas, adolescentes, imunogenicidade, segurança, dengue, México

## Abstract

**Objective.:**

To describe the immunogenicity and safety of a tetravalent dengue vaccine (TAK-003) in healthy adolescents living in Mexico City, an area considered non-endemic for dengue (NCT03341637).

**Methods.:**

Participants aged 12–17 years were randomized 3:1 to receive two doses (Month 0 and Month 3) of TAK-003 or placebo. Immunogenicity was assessed by microneutralization assay of dengue neutralizing antibodies at baseline, Months 4 and 9. Solicited and unsolicited adverse events (AEs) were recorded after each vaccination. Serious (SAEs) and medically-attended AEs (MAAEs) were recorded throughout the study.

**Results.:**

400 adolescents were enrolled, 391 (97.8%) completed the study. Thirty-six (9%) were baseline seropositive to ≥1 serotypes (reciprocal titer ≥10). Geometric mean titers (GMTs) in baseline seronegative TAK-003 recipients were 328, 1743, 120, and 143 at Month 4, and 135, 741, 46, and 38 at Month 9 against DENV-1, -2, -3, and -4, respectively. Placebo GMTs remained <10. Tetravalent seropositivity rates in vaccine recipients were 99.6% and 85.8% at Months 4 and 9, respectively. One MAAE in each group was considered treatment-related (TAK-003: injection-site erythema, and placebo: pharyngitis).

**Conclusion.:**

TAK-003 was immunogenic against all four serotypes and was well tolerated in dengue-naïve adolescents living in Mexico City.

## RESUMEN

**Objetivo.:**

Describir la inmunogenicidad y la seguridad de una vacuna tetravalente contra el dengue (TAK-003) en adolescentes sanos residentes en Ciudad de México, considerada un área no endémica de dengue (NCT03341637).

**Métodos.:**

Se asignó de manera aleatoria a un grupo de participantes de 12 a 17 años en una proporción 3:1 para que recibieran dos dosis (en el mes 0 y en el mes 3) de la vacuna TAK-003 o de un placebo. Se evaluó la inmunogenicidad mediante un análisis de microneutralización de anticuerpos neutralizantes del virus del dengue al inicio del estudio y en los meses 4 y 9. Se registraron los eventos adversos de notificación solicitada y los referidos por iniciativa propia después de cada vacunación. A lo largo del estudio se registraron los eventos adversos graves y los que requirieron atención médica.

**Resultados.:**

Participaron 400 adolescentes y 391 (97,8%) finalizaron el estudio. 36 adolescentes (9%) fueron seropositivos a ≥1 serotipos (título recíproco ≥10) al inicio del estudio. La media geométrica de los títulos en las personas seronegativas vacunadas con TAK-003 al inicio del estudio fue de 328, 1743, 120 y 143 en el mes 4 y 135, 741, 46 y 38 en el mes 9 en relación con DENV-1, -2, -3 y -4, respectivamente. La media geométrica de los títulos de las personas que recibieron un placebo se mantuvo en <10. Las tasas de seropositividad tetravalente en los vacunados fueron 99,6% y 85,8% a los meses 4 y 9, respectivamente. Se consideró relacionado con el tratamiento un evento adverso con atención médica que tuvo lugar en cada grupo (TAK-003: eritema en el lugar de la inyección; placebo: faringitis).

**Conclusiones.:**

TAK-003 fue inmunogénica ante los cuatro serotipos y bien tolerada en los adolescentes sin exposición previa al dengue que vivían en Ciudad de México.

## RESUMO

**Objetivo.:**

Descrever a imunogenicidade e a segurança de uma vacina tetravalente contra dengue (TAK-003) em adolescentes saudáveis residentes da Cidade do México, área considerada não endêmica para dengue (ClinicalTrials.gov: NCT03341637).

**Métodos.:**

Participantes com idade entre 12 e 17 anos foram randomizados a uma proporção de 3:1 para receber duas doses da vacina TAK-003 ou placebo (no mês 0 e no mês 3). A imunogenicidade foi avaliada pelos títulos de anticorpos neutralizantes contra dengue determinados em ensaio de microneutralização ao início do estudo, no mês 4 e no mês 9. A ocorrência de eventos adversos solicitados ou espontâneos foi registrada após cada rodada de vacinação. Eventos adversos graves e eventos adversos que exigiram atendimento médico foram monitorados ao longo de todo o estudo.

**Resultados.:**

De 400 adolescentes incluídos na amostra estudada, 391 (97,8%) completaram o estudo. Trinta e seis (9%) apresentaram positividade basal a um ou mais sorotipos virais da dengue (título recíproco ≥10). A média geométrica dos títulos de anticorpos nos vacinados com TAK-003 que eram soronegativos ao início do estudo foi de 328, 1743, 120 e 143 no mês 4 e 135, 741, 46 e 38 no mês 9, contra os sorotipos virais DENV-1, DENV-2, DENV-3 e DENV-4, respectivamente. A média geométrica dos títulos de anticorpos no grupo placebo se manteve abaixo de 10. A taxa de soropositividade tetravalente nos vacinados foi de 99,6% no mês 4 e 85,8% no mês 9. Um único evento adverso que exigiu atendimento médico em cada grupo foi considerado relacionado ao tratamento (eritema no local de aplicação no grupo TAK-003 e faringite no grupo placebo).

**Conclusão.:**

A vacina TAK-003 demonstrou ser imunogênica contra os quatro sorotipos virais da dengue e foi bem tolerada em adolescentes residentes da Cidade do México sem história pregressa de infecção pela dengue.

Preparation for epidemics, such as those cause by vector-borne diseases, has been identified by the World Health Organization as one of urgent health challenges for the next decade (1). With mosquito populations spreading into new areas, accelerated by changes in climate, the incidence of dengue fever has been increasing rapidly over recent decades (1, 2). The four dengue virus serotypes (DENV-1, DENV-2, DENV-3, and DENV-4), primarily transmitted by female *Aedes aegypti* mosquitoes, lead to a wide range of clinical manifestations which include life-threatening illnesses (3). Approximately half of the global population lives in areas at risk of dengue transmission, (3) and rates of infection in travelers to endemic areas have increased substantially since the early 1990s (4, 5). Annually, an estimated 96 million symptomatic dengue infections occur worldwide, plus an estimated additional 290 million asymptomatic or mild infections, which are not recorded by national surveillance systems (3).

Dengue accounts for approximately 2% of all febrile illnesses in travelers returning from the tropics (6), and is a more frequent cause of febrile illness than malaria in travelers to Southeast Asia (7, 8). However, the true burden of dengue in travelers is likely to be considerably higher due to variability in reporting, misdiagnoses, and patients not seeking treatment for mild illness (6). For example, prospective seroconversion studies estimated an incidence of 2.9% in Dutch travelers who spent approximately one month in dengue-endemic area of Asia (9), and 6.7% in Israeli travelers to endemic tropical regions for 6 months (10). While currently a disease of tropical areas, the increasing global range of *Aedes aegypti* and *Aedes albopictus* mosquitoes presents a risk for spread of dengue in non-endemic areas (11, 12), and outbreaks have occurred in recent years in a number of non-endemic countries in Europe and North America (13).

CYD-TDV (Dengvaxia^®^, Sanofi Pasteur), a tetravalent dengue vaccine, was first licensed in Mexico in 2015 and is now approved for use in 20 countries worldwide (14). Given the observed increased risk of hospitalized and severe dengue in dengue-naïve (seronegative) vaccine recipients (15, 16), together with a variable vaccine efficacy across serotypes (17), there remains an unmet need for a tetravalent vaccine which is effective regardless of previous dengue exposure and can also provide high level of efficacy across serotypes.

A new tetravalent dengue vaccine candidate, TAK-003 (Takeda), is based on a live attenuated DENV-2 virus that provides the genetic backbone for all four of the viruses in the vaccine, which were originally designed and constructed by scientists at the Division of Vector-Borne Diseases of the Centers for Disease Control and Prevention (CDC) (18). The DENV-2 strain (TDV-2) is based on an attenuated laboratory-derived virus, DEN-2 primary dog kidney (PDK)–53 (19). The other three virus strains (TDV-1, TDV-3, and TDV-4) are chimeras that were generated by replacing the pre-membrane and envelope genes of TDV-2 with those from wild-type DENV-1, DENV-3, and DENV-4 strains (20).

In previous phase 1 and 2 studies, TAK-003 was generally well tolerated and demonstrated immunogenicity in children, adolescents, and adults, irrespective of baseline serostatus (21-25). In a recent phase 2 study in children and adolescents aged 2–17 years, antibody persistence was observed four years after vaccination, with a decreased risk of virologically confirmed dengue (VCD) in vaccine versus placebo recipients (relative risk: 0.35; 95% confidence interval [CI]: 0.19–0.65) (25). In the subset of individuals in whom immunogenicity was assessed, 45% were seronegative at baseline. In the ongoing phase 3 efficacy study in children and adolescents aged 4–16 years living in dengue-endemic areas of Asia and Latin America, vaccine efficacy against VCD caused by any serotype was 80.2% (73.3–85.3%) during the first year after vaccination (26) and 66.2% (49.1–77.5%) during the first one and half years post-vaccination in those who were seronegative at baseline (27). However, efficacy varied by serotype and no efficacy was seen against DENV-3 in baseline seronegatives. Interestingly, 81% efficacy (95% CI: 64.1–90.0%) across serotypes was observed after the first dose in the ~ 3 months period before administration of the second dose (27).

Previous clinical trials of TAK-003 have been conducted in both endemic and non-endemic areas. Across the clinical development program, studies in endemic areas have mostly enrolled children and adolescents, whereas studies in non-endemic areas have so far only been conducted in adults (21-29). Adolescents enrolled in the studies in endemic areas have been predominantly pre-exposed to dengue. We therefore conducted a study in adolescents living in a dengue non-endemic area, as this would provide important safety and immunogenicity data in a population presumed to be predominantly dengue-naïve. While endemic in many parts of Mexico, dengue is not endemic in Mexico City due to the high altitude (30). In this phase 3 randomized placebo-controlled study, we assessed the immunogenicity and safety of two doses of TAK-003 in healthy adolescents aged 12–17 years living in Mexico City. To understand the clinical relevance of the immune response and to investigate any unexpected immunological differences between populations (e.g. due to exposure to other flaviviruses), the findings from this study were descriptively compared with those from baseline seronegative adolescents (12–16 years of age) in Latin America enrolled in the ongoing phase 3 efficacy study (NCT02747927).

The objective of this study was to describe the immunogenicity and safety of TAK-003 in dengue-naive adolescents living in Mexico City, an area considered non-endemic for dengue.

## METHODS

This phase 3 randomized, double-blind, placebo-controlled study was performed at five sites in Mexico City between December 2017 and January 2019. Healthy adolescents aged 12–17 years were eligible for enrolment. Main exclusion criteria included hypersensitivity/allergy to any vaccine component; febrile illness at enrolment (≥38°C); serious or chronic progressive disease; impaired/altered immune function; body mass index ≥ 35kg/m^2^; pregnancy or breastfeeding; receipt of other vaccines within 14 days (inactivated) or 28 days (live vaccines) prior to first visit; participation in another clinical trial within 30 days of the first visit; or previous vaccination against or history of infection with dengue or any other flavivirus.

Participants were randomized 3:1 using an interactive web response system to receive either two doses of TAK-003 three months apart (administered at Month 0 [Day 1] and Month 3 [Day 91]), or placebo. All participants were followed for six months following administration of the second dose, leading to a total study duration of approximately nine months.

TAK-003 vaccine was provided as a lyophilized formulation which was reconstituted with saline prior to subcutaneous injection (needle length: 25 G x 1”) preferentially into the deltoid muscle of the non-dominant arm. A single 0.5 mL dose of TAK-003 (lot number: PPQ0010617) contained approximately 5.1, 4.5, 5.4, and 5.9 log_10_ plaque-forming units of TDV-1, TDV-2, TDV-3, and TDV-4, respectively. Normal saline for injection was used as placebo. TAK-003, diluent, and placebo were shipped in refrigerated containers and stored at 2 °C to 8 °C until use.

Blood samples (5 mL) were taken for immunogenicity evaluations at baseline and at Months 4 and 9. Immunogenicity was assessed as geometric mean titers (GMTs) of dengue neutralizing antibodies using a microneutralization assay, with titers corresponding to the dilution which resulted in a 50% plaque reduction (MNT_50_) (31). The primary study objective was assessment of the neutralizing antibody response against each dengue serotype at one month after the second dose of TDV or placebo (Month 4). Secondary immunogenicity objectives included persistence of antibody titers to Month 9, and assessment of seropositivity rates against individual and multiple dengue serotypes at Months 4 and 9. Seropositivity was defined as a reciprocal neutralizing titer ≥10. Participants were assessed for seropositivity at baseline, with seronegativity being defined as absence of seropositivity to any of the four dengue serotypes.

For safety outcomes, solicited local (injection site pain, erythema, and swelling) and systemic (headache, malaise, myalgia, asthenia, and fever ≥38 °C) adverse events (AEs) were recorded on diary cards for seven and 14 days, respectively, following each vaccination. Unsolicited AEs were monitored for 28 days following each vaccination. Serious (SAEs) and non-serious medically-attended (MAAEs) AEs were monitored throughout the study. AEs were graded for severity (mild, moderate, or severe) and causal relationship to the study vaccine or procedures was assessed by the study investigator.

This was a descriptive study and all the analyses planned were therefore descriptive in nature. Hence, no formal statistical hypotheses were planned to be tested in this study. The sample size was not determined by any formal statistical power calculations but was considered sufficient to address the study objectives. The randomization of 3:1 was chosen to allow a higher proportion of participants to receive TAK-003. Immunogenicity data are presented as GMTs and associated 95% confidence intervals for the per-protocol set (PPS), i.e. all participants who were seronegative at baseline, received at least one dose of TAK-003 or placebo, and had no major protocol deviations. Antibody titers below the lower limit of detection (LLOD) were imputed with a value of five (half the LLOD).

GMTs and seropositivity rates were descriptively compared with those from the baseline seronegative adolescent Latin America population from the pivotal phase 3 efficacy study. In that ongoing trial in eight dengue endemic countries of Asia and Latin America, 20 099 healthy 4–16 year old children and adolescents were randomized in the ratio of 2:1 to receive two doses of TAK-003 or placebo three months apart. The trial has multi-year post-vaccination follow up to detect symptomatic dengue to demonstrate efficacy, safety and immunogenicity of TAK-003. The trial plan included assessment of baseline serostatus of all participants and periodic immunogenicity assessment in a randomly selected subset of participants over a longer term. Full details of the phase 3 efficacy study design have been published previously (26). This study was chosen as a comparison as it contained the largest population of seronegative adolescents from any of the TAK-003 studies to date, and this group was chosen as the most similar demographic to that of the current study population. This comparison was performed as a post-hoc analysis between these two studies. Safety data are presented for the safety set, i.e. all participants who received at least one dose of TAK-003 or placebo. All analysis was performed using SAS 9.4 (SAS Institute Inc 2013. Cary, NC: SAS Institute Inc.).

This study was performed in compliance with the Declaration of Helsinki and the principles of Good Clinical Practice (GCP). Written informed consent was obtained from parents/legal guardians of all participants prior to enrolment in the study. Informed assent was also obtained from participants. The protocol, protocol amendments, informed assent/consent forms, and other relevant material were approved by the institutional review boards and ethics committees prior to commencement of the study. The study is registered at clinicaltrials.gov (NCT03341637).

## RESULTS

**FIGURE 1.**
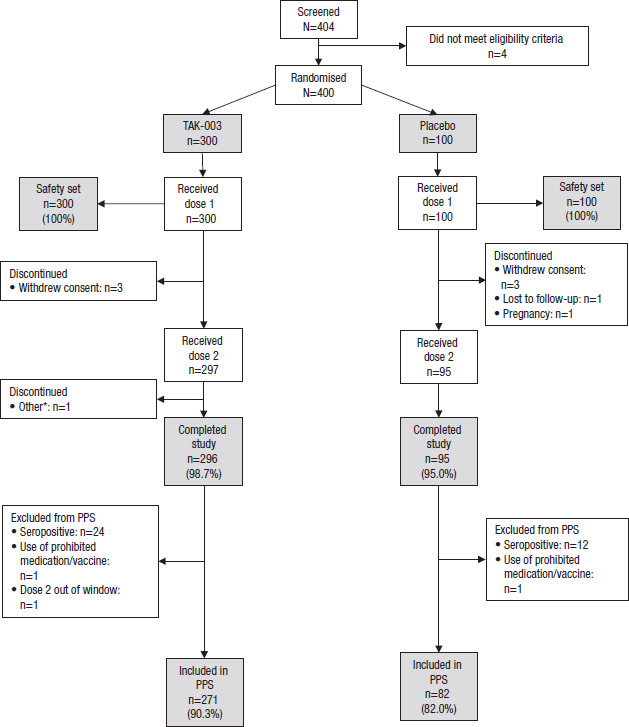
Study participation flowchart from screening to study completion, including reasons for non-randomization, discontinuation, and exclusion from the per protocol set

Of the 400 participants who were enrolled, 296 out of 300 (98.7%) in the TAK-003 group and 95 out of 100 (95.0%) in the placebo group completed the study (Figure 1). In total, 36 participants (24 in the TAK-003 group, and 12 in the placebo group) were seropositive for at least one dengue serotype at baseline and were excluded from the PPS.

### Baseline characteristics

Participant demographics and baseline characteristics were similar across the two study groups. All participants were Hispanic or Latino, and the mean age was 14.3 years in both the PPS and safety sets. Approximately half of participants were taking concomitant medications, and 42–43% had concurrent medical conditions. The most common concomitant medications were analgesics (33% in the TAK-003 group, 27% in the placebo group), and the most common concurrent medical conditions were metabolism and nutrition disorders (11% in the TAK-003 group, 10% in the placebo group).

### Immunogenicity

**FIGURE 2.**
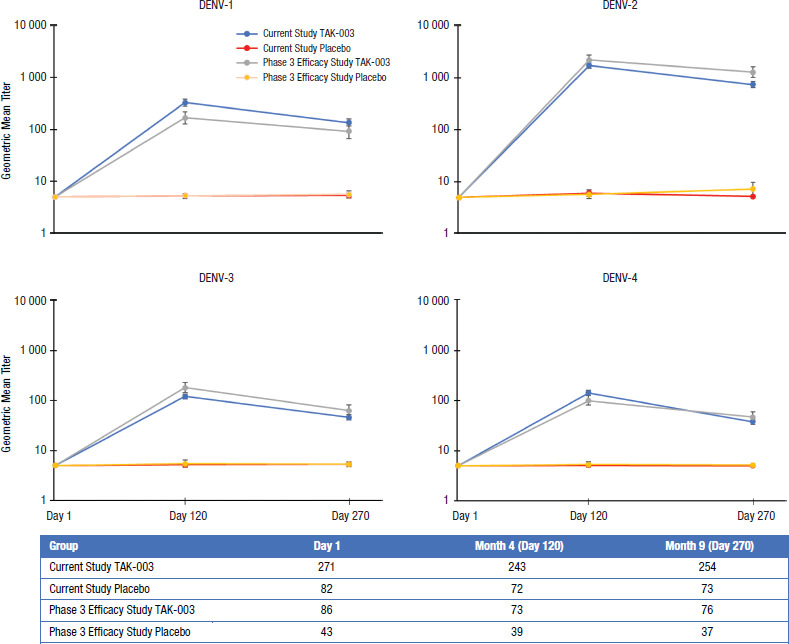
Geometric mean titres (GMTs) of dengue neutralizing antibodies (microneutralization assay) and 95% confidence intervals against each serotype in the study (“current study”), and from seronegative adolescents in Latin America enrolled in a separate phase 3 efficacy study (“Phase 3 efficacy study”). Per protocol set data

By Month 4 (one month after the second vaccination), GMTs had increased against all four serotypes in the TAK-003 group, and remained high through to the end of the study, six months after receipt of the second dose (Figure 2). In TAK-003 recipients, GMTs by Month 4 were 328 (95% CI: 282–382), 1 743 (1 523–1 994), 120 (106–134), and 143 (126–161), and by Month 9 were 135 (115–159), 741 (645–851), 46 (41–52), 38 (33–43) against DENV-1, DENV-2, DENV-3, and DENV-4, respectively. GMTs in the placebo group remained around baseline levels throughout the study.

Of the 3 993 participants included in the subset for immunogenicity assessments in the phase 3 efficacy study, 1 109 (27.8%) were baseline seronegative (26). In total, 129 participants (86 in the TAK-003 group, 43 in the placebo group) were in the population of seronegative adolescents in Latin America in the per protocol immunogenicity subset. GMTs in vaccine recipients in this population were 167 (127–220), 2 194 (1 776–2 710), 181 (143–230), and 100 (81–125) at Month 4 and 92 (66–128), 1 285 (1 021–1 619), 63 (49–81), and 47 (37–60) at Month 9 against DENV-1, -2, -3, and -4, respectively (Figure 2). GMTs in the placebo group remained around baseline levels. GMTs against DENV-2 and -3 in the TAK-003 group were numerically higher than in the current study at both timepoints, whereas they were numerically lower against DENV-1. For DENV-4, the GMTs were numerically lower than the current study at Month 4, whereas they were numerically higher at Month 9. In view of the variability of antibody titers observed in MNT assay, the titers against individual serotypes observed in both these populations could be considered similar.

**FIGURE 3.**
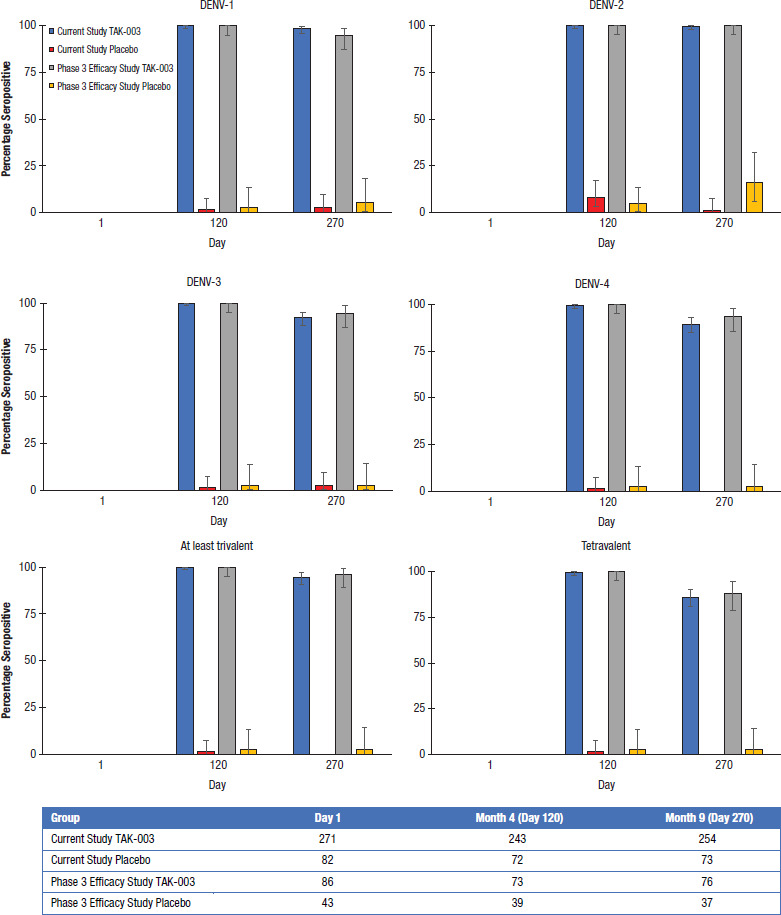
Seropositivity rates and 95% confidence intervals of dengue neutralising antibodies (measured by microneutralization assay) against individual and multiple serotypes in the study (“current study”), and in seronegative adolescents in Latin America enrolled in a separate phase 3 efficacy study (“Phase 3 efficacy study”). Per protocol set data

Post-vaccination seropositivity rates against individual serotypes and multiple serotypes (Figure 3) were high in the TAK-003 group at both timepoints. Seropositivity rates ranged from 99.6%–100% at Month 4, and 89.4%–99.6% at Month 9 across serotypes. Tetravalent seropositivity rates were high in vaccine recipients; 99.6% (97.7–100.0%) at Month 4, and 85.8% (80.9–89.9%) at Month 9. Seropositivity rates were also high in seronegative adolescents in Latin America in the phase 3 efficacy study, where 100% (95.1–100.0%) of TAK-003 recipients had tetravalent seropositivity at Month 4, and 88.2% (78.7–94.4%) at Month 9. Rates against individual serotypes were also in the same range as the current study (Figure 3).

*Other: Participant received second dose outside of scheduled window

PPS, per protocol set, i.e. all participants who were seronegative at baseline, received at least one dose of TAK-003 or placebo, and had no major protocol deviations

Source: Figure prepared by the authors from current study data

### Safety

No deaths or AEs leading to withdrawal were reported during the study. Four SAEs were reported by three participants during the study: two were reported by two participants in the placebo group (both moderate and after the second vaccination; appendicitis and ankle fracture) and two by one participant in the TAK-003 group (both severe and after the first vaccination; abdominal pain and urinary tract infection). None of the SAEs was related to the trial vaccination or trial procedures, and none led to trial vaccination withdrawal or trial discontinuation.

Rates of unsolicited AEs within 28 days after any vaccination were 43.3% in the TAK-003 group and 38.0% in the placebo group (Table 1). In both groups, the most commonly reported AE classified by MedDRA system organ class was infections and infestations. Three participants in the TAK-003 group and none in the placebo group reported rash. Seventeen unsolicited AEs considered related to the study vaccine were reported by eleven participants in the TAK-003 group (3.7%) and one was reported in the placebo group (1.0%). These were gastrointestinal disorders (1% of participants); asthenia (0.3%); injection site pain (1%), erythema (0.3%), or swelling (0.3%); dizziness (1%); epistaxis (1%); rash (0.3%), and maculo-papular rash (0.3%) in the TAK-003 group, and one case of pharyngitis in the placebo group. MAAEs were reported by 47.3% of participants in the TAK-003 group and 38.0% in the placebo group over the entire course of the study. One participant in each group reported an MAAE which in a blinded evaluation was judged by the investigator as being potentially related to the study vaccination (reported in related unsolicited AEs above).

Number of participants with available data within protocol-specified visit window for each time point are given in the table below the figure

Immunogenicity data are presented for the per-protocol set (PPS), i.e. all participants who were seronegative at baseline, received at least one dose of TAK-003 or placebo, and had no major protocol deviations.

Geometric mean titers of dengue neutralizing antibodies were assessed using a microneutralization assay, with titers corresponding to the dilution which resulted in a 50% plaque reduction (MNT_50_). Antibody titers below the lower limit of detection (LLOD) were imputed with a value of five (half the LLOD).

Seropositivity was defined as a reciprocal neutralizing titer ≥10.Seronegativity was defined as absence of seropositivity to any of the four dengue serotypes

Source: Figure prepared by the authors from current study and the phase 3 efficacy study data.

Overall, 70.9% of participants in the TAK-003 group and 49.5% in the placebo group reported solicited local AEs after any vaccination (Table 2). The most frequent local AE was injection site pain, reported by 70.2% in the TAK-003 group and 49.5% in the placebo group. Nearly all the local AEs were mild to moderate in severity. Rates of solicited systemic AEs (Table 3) were 74.6% in the TAK-003 group and 67.7% in the placebo group. The most frequently reported solicited systemic AE in both groups was headache (TAK-003: 56.9%; placebo: 53.5%). Severe systemic AEs were reported by 8.4% of participants in the TAK-003 group and 7.1% in the placebo group. Similar rates of local and systemic AEs were reported after the first versus the second vaccination in both study groups.

Number of participants with available data within protocol-specified visit window for each time point are given in the table below the figure

Immunogenicity data are presented for the per-protocol set (PPS), i.e. all participants who were seronegative at baseline, received at least one dose of TAK-003 or placebo, and had no major protocol deviations.

Seropositive was defined as a reciprocal neutralizing titer ≥10

Source: Figure prepared by the authors from current study and the phase 3 efficacy study data

**TABLE 1.** Most frequently reported unsolicited adverse events in the study (>2% in either treatment group) up to 28 days following any vaccination (first dose at Month 0 or second dose at Month 3) by MedDRA system organ class. Safety set data.

System Organ Class / Preferred Term	TAK-003 (N=300)		Placebo (N=100)	
	Events	Participants (%)	Events	Participants (%)
Any Adverse Events	194	130 (43.3)	50	38 (38.0)
Gastrointestinal disorders	25	22 (7.3)	4	4 (4.0)
General disorders and administration site conditions	12	9 (3.0)	2	2 (2.0)
Infections and infestations	94	80 (26.7)	35	30 (30.0)
Viral upper respiratory tract infection	26	25 (8.3)	10	9 (9.0)
Nasopharyngitis	14	14 (4.7)	4	4 (4.0)
Viral pharyngitis	7	7 (2.3)	6	6 (6.0)
Pharyngitis	8	8 (2.7)	3	3 (3.0)
Injury, poisoning and procedural complications	12	12 (4.0)	0	0
Musculoskeletal and connective tissue disorders	8	8 (2.7)	0	0
Nervous system disorders	12	10 (3.3)	1	1 (1.0)
Respiratory, thoracic and mediastinal disorders	9	7 (2.3)	6	4 (4.0)
Skin and subcutaneous tissue disorders	8	8 (2.7)	1	1 (1.0)

Source: Table prepared by the authors from current study data

**TABLE 2.** Number of participants (%) in the study reporting solicited local adverse events (AEs) occurring up to seven days after each vaccination at Months 0 and 3. Safety set data

AE severity	TAK-003			Placebo		
	Any vaccination (n=299)^a^	First vaccination (n=299)	Second vaccination (n=295)^b^	Any vaccination (n=99)^a^	First vaccination (n=99)	Second vaccination (n=94)^b^
**Any solicited local AE**						
Any	212 (70.9)	168 (56.2)	154 (52.2)	49 (49.5)	34 (34.3)	29 (30.9)
Mild	145 (48.5)	137 (45.8)	106 (35.9)	37 (37.4)	26 (26.3)	23 (24.5)
Moderate	56 (18.7)	29 (9.7)	39 (13.2)	10 (10.1)	7 (7.1)	5 (5.3)
Severe	11 (3.7)	2 (0.7)	9 (3.1)	2 (2.0)	1 (1.0)	1 (1.1)
**Pain**						
Any	210 (70.2)	165 (55.2)	153 (51.9)	49 (49.5)	34 (34.3)	29 (30.9)
Mild	143 (47.8)	134 (44.8)	105 (35.6)	37 (37.4)	26 (26.3)	23 (24.5)
Moderate	56 (18.7)	29 (9.7)	39 (13.2)	10 (10.1)	7 (7.1)	5 (5.3)
Severe	11 (3.7)	2 (0.7)	9 (3.1)	2 (2.0)	1 (1.0)	1 (1.1)
**Erythema**						
Any	25 (8.4)	17 (5.7)	12 (4.1)	0	0	0
Mild: 2.5-5 cm	25 (8.4)	17 (5.7)	12 (4.1)	0	0	0
**Swelling**						
Any	17 (5.7)	13 (4.3)	6 (2.0)	0	0	0
Mild: 2.5-5 cm	16 (5.4)	13 (4.3)	5 (1.7)	0	0	0
Moderate: >5-≤10 cm	1 (0.3)	0	1 (0.3)	0	0	0

Severity categories are excluded from the table if no participants in either study group experienced AEs after either of the vaccinations

“Any vaccination” refers to the number of participants reporting AEs after either of the vaccinations

a One participant in each study group did not provide a diary card

b Only includes participants who received the second vaccination and provided completed diary cards

Source: Table prepared by the authors from current study data

## DISCUSSION

This phase 3 study assessed the immunogenicity and safety of a two-dose primary schedule of TAK-003 versus placebo in seronegative adolescents living in an area considered non-endemic for dengue. Overall, 364 of the 400 participants (91%) were seronegative at baseline, confirming the assumption of a high proportion of dengue-naïve adolescents in Mexico City. GMTs increased in vaccine recipients post-vaccination, persisted to six months after vaccination, and were generally similar in magnitude and pattern to those observed in a group of seronegative adolescents in Latin America enrolled in the ongoing phase 3 efficacy study. No changes in GMTs were observed in the placebo group. Seropositivity rates in vaccine recipients were high against individual and multiple serotypes, and were consistent with those reported in the phase 3 efficacy study. No important new safety issues were identified.

**TABLE 3.** Number of participants (%) in the study reporting solicited systemic adverse events (AEs) occurring up to fourteen days after each vaccination at Months 0 and 3. Safety set data

AE severity	TAK-003			Placebo		
	Any vaccination (N=299)^a^	First vaccination (N=299)	Second vaccination (N=296)^b^	Any vaccination (N=99)^a^	First vaccination (N=99)	Second vaccination (N=94)^b^
**Any solicited systemic AE**						
Any^c^	223 (74.6)	202 (67.6)	150 (50.7)	67 (67.7)	58 (58.6)	43 (45.7)
Mild	127 (42.5)	134 (44.8)	93 (31.4)	32 (32.3)	32 (32.3)	27 (28.7)
Moderate	67 (22.4)	53 (17.7)	38 (12.8)	28 (28.3)	22 (22.2)	12 (12.8)
Severe	25 (8.4)	12 (4.0)	15 (5.1)	7 (7.1)	4 (4.0)	4 (4.3)
**Headache**						
Any	170 (56.9)	133 (44.5)	105 (35.5)	53 (53.5)	44 (44.4)	28 (29.8)
Mild	112 (37.5)	94 (31.4)	72 (24.3)	28 (28.3)	25 (25.3)	19 (20.2)
Moderate	43 (14.4)	31 (10.4)	25 (8.4)	22 (22.2)	18 (18.2)	6 (6.4)
Severe	15 (5.0)	8 (2.7)	8 (2.7)	3 (3.0)	1 (1.0)	3 (3.2)
**Asthenia**						
Any	137 (45.8)	104 (34.8)	83 (28.0)	44 (44.4)	35 (35.4)	26 (27.7)
Mild	92 (30.8)	74 (24.7)	59 (19.9)	27 (27.3)	20 (20.2)	21 (22.3)
Moderate	35 (11.7)	23 (7.7)	20 (6.8)	14 (14.1)	13 (13.1)	4 (4.3)
Severe	10 (3.3)	7 (2.3)	4 (1.4)	3 (3.0)	2 (2.0)	1 (1.1)
**Malaise**						
Any	118 (39.5)	83 (27.8)	70 (23.6)	42 (42.4)	33 (33.3)	22 (23.4)
Mild	73 (24.4)	56 (18.7)	48 (16.2)	22 (22.2)	20 (20.2)	12 (12.8)
Moderate	34 (11.4)	23 (7.7)	15 (5.1)	15 (15.2)	11 (11.1)	6 (6.4)
Severe	11 (3.7)	4 (1.3)	7 (2.4)	5 (5.1)	2 (2.0)	4 (4.3)
**Muscle pain (myalgia)**						
Any	165 (55.2)	143 (47.8)	103 (34.8)	50 (50.5)	40 (40.4)	29 (30.9)
Mild	112 (37.5)	108 (36.1)	76 (25.7)	30 (30.3)	26 (26.3)	20 (21.3)
Moderate	44 (14.7)	32 (10.7)	20 (6.8)	18 (18.2)	13 (13.1)	8 (8.5)
Severe	9 (3.0)	3 (1.0)	7 (2.4)	2 (2.0)	1 (1.0)	1 (1.1)
**Fever (°C)**						
Any	38 (12.7)	20 (6.7)	20 (6.8)	8 (8.1)	5 (5.1)	3 (3.2)
38.0-<38.5	18 (6.0)	9 (3.0)	11 (3.7)	4 (4.0)	3 (3.0)	1 (1.1)
38.5-<39.0	13 (4.3)	7 (2.3)	6 (2.0)	3 (3.0)	2 (2.0)	1 (1.1)
39.0-<39.5	5 (1.7)	3 (1.0)	2 (0.7)	1 (1.0)	0	1 (1.1)
39.5-<40.0	2 (0.7)	1 (0.3)	1 (0.3)	0	0	0

“Any vaccination” refers to the number of participants reporting AEs after either of the vaccinations

a One participant in each study group did not provide a diary card

b Only includes participants who received the second vaccination and provided completed diary cards

c Fever is included in the “any” category but was not assessed by severity (mild/moderate/severe)

Source: Table prepared by the authors from current study data

This was the first study of TAK-003 specifically designed to assess responses to TAK-003 in seronegative adolescents in a non-endemic setting. As previous studies of TAK-003 immunogenicity and safety have focused predominantly on endemic areas (24, 25, 28, 32, 33), or in dengue-naïve adults in non-endemic areas (21-23, 29), this study contributes important data on the vaccine effects in dengue-naïve adolescents for whom there is no currently recommended vaccine. Consistent with all previous studies of TAK-003, highest GMTs were observed against DENV-2 (24-26, 28, 32, 33), which also corresponded with the highest efficacy against this serotype observed in the ongoing phase 3 efficacy study (27). While efficacy was not assessed in the current study, the similarities between the antibody responses seen in this study and those from the phase 3 efficacy study could provide guidance on the potential efficacy in adolescents for travel vaccination. Despite the absence of a correlate of protection as a surrogate of vaccine efficacy, and given the practical prohibitions of undertaking efficacy trials in travelers, immunogenicity comparison may be a practical way of providing insight into how the vaccine may perform across populations.

In the phase 3 efficacy study, the overall vaccine efficacy in adolescents (12–16 years of age) including all serotypes was 82.0% (95%CI: 70.0–89.2) during the first one and a half years post-vaccination. Across all seronegative participants, the vaccine efficacy was 67.8% (95% CI: 40.3–82.6%) and 98.1% (85.8–99.7%) against DENV-1 and DENV-2, respectively; no efficacy was observed against DENV-3; and there were too few DENV-4 cases for assessment (27). Additionally, the observed rapid onset of protection after the first dose is also relevant in this context. Further data on DENV-3 and DENV-4 in seronegative individuals, and the results from the long term follow up in the ongoing phase 3 efficacy trial will be important to better define this vaccine profile, particularly for potential use in travelers.

The safety findings in this study were consistent with reports from previous studies and the vaccine was well tolerated. The majority of solicited and unsolicited AEs were mild to moderate, with injection site pain and headache being the most frequently reported local and systemic AEs, respectively, as observed previously (22, 23, 28, 29). Rash was infrequent and was reported by only a few participants.

One limitation of this study was the relatively short follow-up duration for assessment persistence of the immune response. Although long-term antibody persistence to TAK-003 was recently shown in a four-year phase 2 study in children and adolescents in dengue-endemic areas and will be further assessed in the ongoing phase 3 efficacy trial, there may be differential persistence in endemic versus non-endemic regions due to natural dengue exposure (25). As a mitigation, eligible and willing participants from this trial, along with those from another trial that evaluated TAK-003 in adults in the United States, will be evaluated in a follow up study to assess antibody persistence over a longer time period (NCT03999996). A booster dose is also planned to be evaluated in the same study.

In summary, this study provides important data on the effects of TAK-003, a tetravalent dengue vaccine, in dengue-naïve adolescents. These data will aid evaluation of the potential use of this vaccine for people living in and travelling to dengue endemic areas. TAK-003 was immunogenic against all four serotypes and was well tolerated in dengue-naïve adolescents living in Mexico City.

## Disclaimer.

Authors hold sole responsibility for the views expressed in the manuscript, which may not necessarily reflect the opinion or policy of the RPSP/PAJPH and/or PAHO
